# Predicting hospital mortality among frequently readmitted patients: HSMR biased by readmission

**DOI:** 10.1186/1472-6963-11-57

**Published:** 2011-03-14

**Authors:** Wim F van den Bosch, Johannes C Kelder, Cordula Wagner

**Affiliations:** 1 St. Antonius Hospital, P.O. Box 2500, 3430 EM Nieuwegein, the Netherlands; 2 NIVEL, the Netherlands Institute for Health Services Research, P.O. Box 1568, Utrecht, the Netherlands; 3 VU University Medical Centre, De Boelelaan 1117, 1081 HV Amsterdam, the Netherlands

## Abstract

**Background:**

Casemix adjusted in-hospital mortality is one of the measures used to improve quality of care. The adjustment currently used does not take into account the effects of readmission, because reliable data on readmission is not readily available through routinely collected databases. We have studied the impact of readmissions by linking admissions of the same patient, and as a result were able to compare hospital mortality among frequently, as opposed to, non-frequently readmitted patients. We also formulated a method to adjust for readmission for the calculation of hospital standardised mortality ratios (HSMRs).

**Methods:**

We conducted a longitudinal retrospective analysis of routinely collected hospital data of six large non-university teaching hospitals in the Netherlands with casemix adjusted standardised mortality ratios ranging from 65 to 114 and a combined value of 93 over a five-year period. Participants concerned 240662 patients admitted 418566 times in total during the years 2003 - 2007. Predicted deaths by the HSMR model 2008 over a five-year period were compared with observed deaths.

**Results:**

Numbers of readmissions per patient differ substantially between the six hospitals, up to a factor of 2. A large interaction was found between numbers of admissions per patient and HSMR-predicted risks. Observed deaths for frequently admitted patients were significantly lower than HSMR-predicted deaths, which could be explained by uncorrected factors surrounding readmissions.

**Conclusions:**

Patients admitted more frequently show lower risks of dying on average per admission. This decline in risk is only partly detected by the current HSMR. Comparing frequently admitted patients to non-frequently admitted patients commits the constant risk fallacy and potentially lowers HSMRs of hospitals treating many frequently admitted patients and increases HSMRs of hospitals treating many non-frequently admitted patients. This misleading effect can only be demonstrated by an analysis over a prolonged period, but occurs, in effect, every day of the year. This finding is relevant for all countries where hospitals use HSMR for monitoring and improving hospital performance. The use of 'admission frequency' as additional adjustment variable may provide a more accurate HSMR.

## Background

In various countries in the world, risk adjusted in-hospital mortality ratios are currently calculated using routinely collected data. The variations seen in crude mortality for which adjustment is needed, may be attributed to various sources, including variations in: registration data, casemix, quality of care and chance [1]. After adjustment for casemix, the hospital standardised mortality ratio (HSMR) can be used as a tool for hospitals to analyse their death rates by comparing their riskadjusted mortality with the national average, establishing a starting point for improvement of hospital outcomes [2]. In some countries HSMRs are also made publicly available for the benefit of for example: the patient, the politician, the hospital manager and the clinician. This public reporting potentially may result in league tables, suggesting that the ranking reflects differences in quality of care. A somewhat more subtle approach is being applied in the UK by combining standardised mortality ratios with other safety indicators, in order to establish hospital safety rankings. These rankings are publicised in the "How Safe is Your Hospital" guide [3], compiled by researchers at the Dr Foster Intelligence Unit at Imperial College London. However, publicly comparing and judging hospital performances in this way is heavily disputed [4,5] at the moment. Some publications mention the Netherlands as one of the countries where HSMRs are also made publicly available [6,7], yet up to and including 2008 the HSMRs have not been publicised with the exception of a few hospitals that voluntarily put their values on the web. Mandatory publication was planned to start in 2010, based on admission data from 2009, and various articles suggest that the quality of the Dutch HSMRs is sufficiently high to justify publication. Heijink et al [8] for example state that they did not find evidence that the HSMR cannot be used as an indicator to monitor and compare hospital quality in the Netherlands. However there is some controversy around this subject and the Dutch Ministry of Health recently decided to postpone mandatory publication for at least one year, because there were doubts about the reliability and validity of the current figure [9]. This decision was based on the outcomes of a study [10] conducted by Santeon, a group of six large non-university teaching hospitals in the Netherlands (Additional file 1: Table S1), confirming indeed why publicly comparing HSMRs may not be a good idea [4].

One of the findings of this Dutch study [10], not being addressed in [4], showed large differences between the hospitals with respect to numbers of readmissions per patient, if measured over a prolonged period of time. Currently the HSMR in the Netherlands is not adjusted however for any form of readmission. In the UK the HSMR is adjusted for readmission for acute cases, but not for a prolonged period of time.

The association between readmissions and the HSMR is mentioned in a paper by Jarman [11]. The effects of adjusting for readmissions are described as follows: *" ....There is also not much difference between normal HSMRs based on all admissions and those based on only one (e.g., the last) admission in a year. ...."*. We interpret this finding as follows: If a patient visits the hospital more than once that year, then any one of the admissions would represent the contribution of that patient to the HSMR ratio. Using a limited part of the patient's admission history, would, on average, not make a difference to the HSMR of that hospital. This may suggest that adjustment for readmission would not make sense. Using this method, we question whether a period of one-year is sufficiently long to embrace all the effects of readmission and indeed whether all the risk conditions surrounding readmissions can be properly addressed. A more recent article by Jarman [2] however, does address the need to investigate adjustment for readmissions as follows: " ... *further improvements to the case-mix model are being evaluated. The numbers of previous admissions within a given time period, which requires the linking of admissions of the same patient, could be of potential use..*..". In our publication we will share how differences in numbers of previous admissions made an impact upon the HSMRs of the hospitals and what can be done to improve the HSMR model used.

In order to allow comparisons to be unbiased, adjustment for the differing risks of patient specific variables, that is the casemix, is necessary. Risk factors used in the adjustment may be related in different ways to the in-hospital risks. Ignoring non-constant risk relationships commits the constant risk fallacy. Attributing the residual (unexplained) variation from case-mix adjusted mortality to quality of care commits the "case-mix adjustment fallacy" [1,12]. An example of this phenomenon applied to the HSMR model in the UK is described by Mohammed et al [6]. Here the interaction of two casemix adjustment variables - comorbidity and admission type - with hospitals was considerable. These effects could be explained by differences in clinical coding and admission practices across hospitals rather than by differences in the quality of care. The lesson we learn from this study is that variables being adjusted may mean different things to different hospitals. Since adjustment of variables is 'admission-based', one can ask whether the practice of admitting and readmitting in itself may be prone to the constant risk fallacy as well. In other words, does an admission of a patient mean the same thing to one hospital as it means to another hospital admitting 'the same patient'? More specifically attuned to our study: are the risk conditions the same for a patient being admitted only once compared to admissions of a frequently admitted patient? In order to analyse this, we addressed the following research questions using HSMRs from six Dutch hospitals:

1. Are there substantial differences in the numbers of readmissions within a given time period between the six hospitals?

2. Is there a significant association between HSMRs and numbers of readmissions per patient?

3. Does the casemix change as readmissions increase and how does it change?

4. How do we adjust for readmission in order to provide more accurate HSMRs and standardised mortality ratios (SMRs) on diagnostic level?

## Methods

### Setting

'Santeon' (Additional file 1: Table S1) is a group of six large non-university teaching hospitals geographically spread over the Netherlands with HSMRs ranging from 65 (favourable) to 114 (poor) over the years 2003 - 2007 and an overall HSMR value of 93 (table 1). This group of cooperating hospitals covers about 10% of the total Dutch hospital healthcare in terms of the number of admissions.

**Table 1:** Admission numbers and HSMR values years 2003 - 2007 Santeon hospitals and overall value.

	Hospital						Overall value
		
	A	B	C	D	E	F	
Number of admissions over period 2003 - 2007	114714	78417	46322	66802	61333	50978	418566

HSMR value over period 2003 - 2007	96	114	82	65	109	94	93

95% Confidence Interval	(93 - 99)	(110 - 118)	(78 - 86)	(62 - 67)	(105 - 113)	(90 - 99)	(91 - 94)

* Hospitals are presented in random order and labelled A - F.

### Statements

1. This study did not concern experimental research, neither research carried out on humans, neither any experimental research on animals.

2. The dataset used in this study concerned a selection of the 'Landelijke Medische Registratie' (National Medical Registration) covering data from the six Santeon hospitals over the years 2003 - 2007. Each of the boards of the six hospitals approved the usage of their part of the dataset.

### HSMR model used in this study

The HSMR of a hospital is based on the predicted risks of death *per admission*. For a certain period of time, for example a year, the HSMR is calculated with the formula:

The Dutch HSMR model 2008 (DHM-2008) used in this study accounts for at least 70% of hospital mortality. Here adjustment is made for: age, sex, admission type (emergency or non-emergency), length of stay, year of discharge, socio-economic deprivation, comorbidity and CCS diagnostic group based on ICD-9 coding. The DHM-2008, was developed by the Dr Foster Unit in the UK in cooperation with 'Prismant' and the 'PraktijkIndex' in the Netherlands. DHM-2008 is very well described in [2] and resembles the UK model. Differences with the UK model concern: the use of day cases (not used in UK) and the use of 50 CCS groups in NL (based on ICD-9 coding) versus 56 CCS groups (based on ICD-10 coding) in UK of which 42 in common. Furthermore the UK model adjusts for palliative care, source of admission and for the number of previous emergency admissions; DHM-2008 does not adjust for any of these three. Calculations of the HSMR-results were carried out by Prismant, an independent research and advisory agency for the Dutch health service.

### Definition of Readmission

The term 'readmission' is often used for unplanned readmissions within a limited period of time, for example 30 days, for treatment of the same disease. Planned readmissions are another frequently occurring form of readmitting patients, particularly for chronically ill patients, for example suffering from neoplasms. In these cases treatment schemes may span a prolonged period of time, even a number of years. Furthermore, a patient may visit the hospital for a different disease than during a previous admission, which in a way can be seen as a readmission as well. In our study we considered all these cases as 'readmissions' of the same patient over the study period of five years. We adopted the term *'n^th ^admission' *[13], according to the definition, applying to admissions that occurred after the 6^th ^admission, where 'n^th^' is an ordinal number - e.g., seventh, eighth, etc. We used the term also for n = 1, 2, ...6.

### Identifying the n^th ^admission of a patient

Routinely collected data on hospital admissions in the Netherlands are being collated in the national medical registration LMR ('Landelijke Medische Registratie'). In general, readmissions are not adequately registered in the LMR. In order to be able to identify the value of n of the n^th ^admission of a patient, we used the dates of discharge combined with the patient's identification number.

### Admission frequency

HSMR-variable data is collected on a 'per admission' basis. The model considers each admission to be an independent stochastic experiment (like repeatedly throwing a dice), separate from previous admissions, for which a risk of death number is predicted and accumulated into the denominator of the HSMR. The admission represents a container or proxy for risk conditions around a single hospitalization of a patient. However, comparing mortality ratios of hospitals for a certain fixed period of time, one might not only be interested in the quality of single admissions, but also in the quality of the *end result *- was the patient discharged alive after the last admission in a row? The patient is the primary subject of interest for hospitals for whom they bear the responsibility for care. For the purpose of this study we introduce a new variable that may serve as a risk proxy more attuned to the patient, taking into account all the patient's admissions during the study period. Since risk conditions may be linked to how often a patient was admitted in total, we allocated each patient to a distinct patient class P(m), where:

Admission frequency m = the number of times a patient was admitted during a fixed period of time

(Occasionally we use the number of times a patient was admitted after the initial admission, in which case we apply the term '*re*admission frequency' = m-1).

The second research question formulated tests the capability of the current HSMR model for predicting mortality accurately for these classes. Based on this we draw conclusions on whether the new variable m turns out to be a valuable addition to the HSMR model. This idea was suggested but, as far as known, not further explored by Jarman [2].

### Admission view versus Patient view

The dataset of all of the 418566 admission records can be grouped in different ways or views. We have studied and compared mortality risks based on the following two different views:

• *Admission view: *this view is based on n^th ^admission classes A(n) where A(1), A(2), etc. represent all admission records for all first admissions, all second admissions, etc. For example each patient in class A(3) contributed one admission record to class A(3), but also one to A(1) and one to A(2).

• *Patient view: *this view is based on admission frequency classes P(m) representing all admission records of patients who have been admitted exactly m times over the five-year period. For example: each patient in class P(3) contributed exactly three admission records to this class and did not contribute any admission record to any other patient view class. In fact, patients with equal admission frequencies are clustered in distinct mutually exclusive classes, in contrast with the admission view where one patient may contribute admission records to many classes. For example every patient contributes one admission record to class A(1).

### Calculating mortality figures

We calculated crude mortality, predicted mortality (based on DHM-2008) and standardised mortality ratios (SMRs) by applying the HSMR formula for each class A(n) and P(m) for m,n = 1, 2, 3, ... In order to preserve power we have grouped the results of the higher (m > 4) classes P(m) as follows: m = 5, 6 into one group, m = 7 - 9 into one group, m = 10 - 20 into one group, and m > 20 into one group. Similarly for A(n). For the SMRs we also calculated the 95% confidence intervals. We analysed the association between observed and predicted outcomes, and goodness of fit for both views.

## Results

During the five-year study period 240662 patients accounted for 418566 admissions in total. 164884 patients (69% of total patients) concerned 'first-and-only' admissions. The other 75778 patients (31% of total patients) who were admitted more than once accounted for 253682 admissions (61% of total admissions) of which 177904 were readmissions (43% of total admissions).

### Variations in admission frequencies

**Figure 1:**
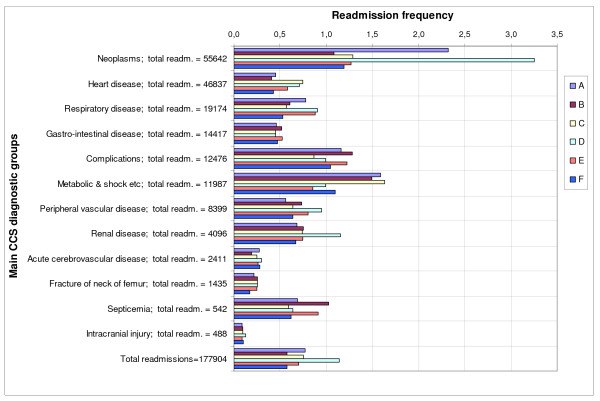
Distribution number of readmissions divided by number of patients per hospital per main CCS diagnostic group *. The chart is showing the average number of readmissions. The six hospitals are labelled A - F. Each diagnostic group title also shows readmission sample sizes, for example there were 55642 readmissions for neoplasms in total.

We analysed the distribution of the admission frequency per patient class P(m) per hospital (table 2). For m = 1 hospital B has the highest percentage admissions (46% of total), hospital D the lowest (29% of total). For P(m > 7) hospital D has high scores, for example: 9% of the admissions concerned patients being admitted more than 20 times (P(m > 20)), whereas for hospitals E and F this percentage amounts to 0,6%. We also analysed the inter-hospital variation of the average admission frequency on the level of the 12 main CCS diagnostic groups (figure 1). The first three diagnostic groups shown - neoplasms, heart diseases and respiratory diseases - cover two thirds of all readmissions. For these diagnostic groups we calculated the ratio between the highest and the lowest observed hospital average of *re*admission frequencies. For neoplasms, hospital D (3.3 readmissions) and hospital B (1.1 readmissions) differed by a factor of 3. For heart diseases and respiratory diseases the ratio between highest and lowest amounted to 1.8 and 1.7 respectively. Overall (bottom line of figure 1) hospital D has the highest average *re*admission frequency (1.14) and hospital B and F have the lowest average (0.57); a factor of 2 difference between the highest and the lowest.

**Table 2:** Distribution of number of (re)admissions over Patient view classes m for each of the six hospitals.

	Percentages of admissions of total admissions per hospital						
	
Patient view class	A	B	C	D	E	F	All hospitals
P(m = 1)	39.1%	46.0%	37.7%	29.3%	38.9%	45.3%	39.4%
P(m = 2)	19.0%	20.8%	20.5%	17.3%	22.4%	21.2%	20.0%
P(m = 3)	10.2%	10.3%	12.3%	10.6%	12.6%	11.2%	11.0%
P(m = 4)	6.2%	6.3%	8.2%	6.6%	7.4%	6.8%	6.8%
P(m = 5, 6)	7.4%	6.5%	9.4%	8.1%	8.8%	7.3%	7.8%
P(m = 7-9)	5.8%	4.6%	5.8%	6.7%	5.2%	4.4%	5.5%
P(m = 10-20)	7.7%	3.9%	4.7%	12.2%	4.2%	3.2%	6.3%
P(m > 20)	4.6%	1.6%	1.5%	9.1%	0.6%	0.6%	3.3%

Total	100%	100%	100%	100%	100%	100%	100%

* Hospitals are presented in random order and labelled A - F.

### Mortality per patient view class and per admission view class

In table 3 the total results of mortality calculations are shown from the perspective of the *patient view *classes. The row of class P(m = 1) shows the outcomes for all 164884 patients who were admitted exactly once, row P(m = 2) for 41876 patients admitted exactly twice, and so on. The crude mortality *per patient *for first-and-only admissions amounts to 5.4%. For m = 2 through to m = 9 we see an average growth in mortality of roughly 1% per patient view class increment. For m = 10 through to 21+ the crude mortality per patient class does not show growth anymore and stabilizes around 13%. Table 3 also shows that DHM-2008 predicts a decline in mortality per admission from 4.2% (P(m = 1)) to 1.1% (P(m > 20)).

**Table 3:** Mortality figures per Patient view class m: crude mortality per patient and per admission, predicted mortality per admission.

	Observed number of			Crude mortality per		DHM-2008 predicted	
	
Patient view class	patients	admissions	deaths	patient	admission	number of deaths	mortality per admission
P(m = 1)	164884	164884	8836	5.4%	5.4%	6971	4.2%
P(m = 2)	41876	83752	2868	6.8%	3.4%	3363	4.0%
P(m = 3)	15356	46068	1377	9.0%	3.0%	1899	4.1%
P(m = 4)	7064	28256	727	10.3%	2.6%	1153	4.1%
P(m = 5, 6)	6055	32490	695	11.5%	2.1%	1302	4.0%
P(m = 7-9)	2938	22795	404	13.8%	1.8%	820	3.6%
P(m = 10-20)	2063	26364	265	12.8%	1.0%	714	2.7%
P(m > 20)	426	13957	55	12.9%	0.4%	157	1.1%

Total	240662	418566	15227	6.3%	3.64%	16379	3.9%

* The numbers represent totals of the six hospitals. The crude mortality per patient stabilises around 13% after 10 (re)admissions. The predicted mortality per admission increasingly differs (higher) from observed mortality for increasing patient view classes. The statistical significance of this is shown in table 5 by means of corresponding standardised mortality ratios and 95% confidence intervals.

In table 4 the results of mortality calculations are shown from the perspective of the *admission view *classes. Row A(n = 1) shows the outcomes for all of the 240662 patients being admitted for the first time. From these patients, 75778 were admitted at least a second time, shown in row A(n = 2), etc. In this case, going from A(n = 1) to A(n > 20), a decline of mortality per admission from 4.1% to 1.1% is predicted by DHM-2008.

**Table 4:** Mortality figures per Admission view class: crude mortality per admission and predicted mortality per admission.

Admission view class	Observed number of		Crude mortality	DHM-2008 predicted	
			
	admissions	deaths	per admission	number of deaths	mortality per admission
A(n = 1)	240662	8836	3.7%	9783	4.1%
A(n = 2)	75778	2868	3.8%	2981	3.9%
A(n = 3)	33902	1377	4.1%	1389	4.1%
A(n = 4)	18546	727	3.9%	740	4.0%
A(n = 5,6)	19124	695	3.6%	714	3.7%
A(n = 7-9)	12634	404	3.2%	421	3.3%
A(n = 10-20)	12483	265	2.1%	293	2.2%
A(n > 20)	5437	55	1.0%	57	1.1%

Total	418566	15227	3.6%	16379	3.9%

* The numbers represent totals of the six hospitals. Predicted mortality per admission is in line with observed mortality for all admission view classes. The statistical significance of this is shown in table 5 by means of corresponding standardised mortality ratios and 95% confidence intervals.

In the patient view the number of observed deaths for first-and-only admissions (P(m = 1)) is clearly higher than predicted and for P(m = 2) - P(m > 20) lower (table 3). In the admission view the differences are smaller (table 4).

**Figure 2:**
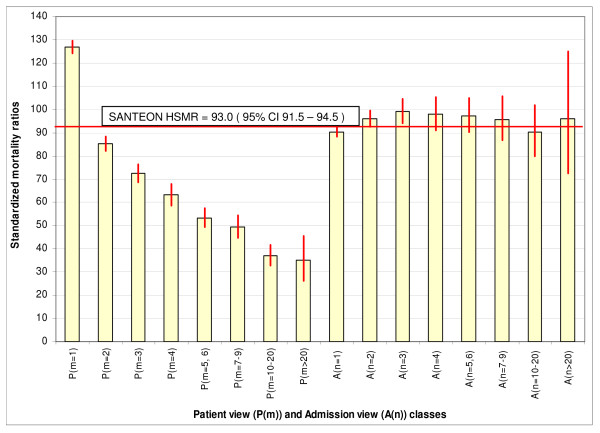
Standardized mortality ratios of Patient view classes and Admission view classes including 95% confidence intervals *. Santeon overall HSMR equals 93.0 (95% CI: 91.5 - 94.5).

From tables 4 and 5 we can calculate the standardised mortality ratios for both views via the ratio of observed deaths and predicted deaths per category. Table 5 shows the results, including 95% confidence intervals (p < 0.0001). Figure 2 presents a graphical representation of this. In the patient view the SMRs decline from 127 (P(m = 1)) to 35 (P(m > 20)) and none of the corresponding 95% confidence intervals includes the expected overall HSMR value 93.0 (95% CI: 91.5 - 94.5). This shows a significant association between patient view categories and SMRs indicating a lack of model fit. In the admission view however, the SMRs fluctuate between 90 and 99 and all corresponding 95% confidence intervals include the overall HSMR value 93.0 (95% CI: 91.5 - 94.5) indicating a good fit of the HSMR model for this view, which we will discuss later.

**Table 5:** Standardised mortality ratios (SMRs) for patient view and for admission view.

**Patient View**	**SMR per Patient View Category**	**Lower 95% CI** **of SMR**	**Upper 95% CI** **of SMR**	**Admission View**	**SMR per Admission View Category**	**Lower 95% CI** **of SMR**	**Upper 95% CI** **of SMR**
	
P(m = 1)	126.8	124.1	129.4	A(n = 1)	90.4	88.5	92.3
P(m = 2)	85.3	82.2	88.5	A(n = 2)	96.0	92.5	99.6
P(m = 3)	72.5	68.7	76.5	A(n = 3)	99.2	94.1	104.6
P(m = 4)	63.1	58.6	67.8	A(n = 4)	98.1	91.1	105.5
P(m = 5, 6)	53.4	49.5	57.5	A(n = 5, 6)	97.4	90.3	104.9
P(m = 7-9)	49.3	44.6	54.3	A(n = 7-9)	95.8	86.7	105.6
P(m = 10-20)	37.1	32.8	41.8	A(n = 10-20)	90.4	79.9	102.0
	
P(m > 20)	34.9	26.3	45.5	A(n > 20)	96.1	72.4	125.1

### Casemix risk profile for the n^th ^admission

Finally, we also studied the variation over the n^th ^admission classes of five casemix variables (table 6). Year, sex and social deprivation are considered less relevant variables for this. We split the table into patient and admission specific property groups. Variations in casemix for n^th ^admissions for n going from 1 to 21+ can be characterized by: an average age that initially increases from 61 (n = 1) to 66 years (n = 4) and then gradually drops to 59 years; a steady increase of comorbidity as indicated by a diminishing contribution of the two lowest and an increasing contribution of the higher Charlson indices; a predominance of heart diseases for lower values of n and a predominance of neoplasms for higher values of n; a decrease of emergency admissions; a decrease of lengths of stay (for n > 3). As readmissions increase the casemix changes as indicated by the combination of variations of these five casemix variables. These changes are associated with a decline in mortality for n > 4 as predicted by DHM-2008 (table 4).

**Table 6:** Values/distributions of major casemix variables for which adjustment is done by DHM-2008 per n^th ^Admission for six hospitals.

	A	A	A	A	A	A	A	A
	(n = 1)	(n = 2)	(n = 3)	(n = 4)	(n = 5,6)	(n = 7-9)	(n = 10-20)	(n > 20)
	
Number of admissions	240662	75778	33902	18546	19124	12634	12483	5437
**Patient specific casemix properties**								

Average age at n^th ^discharge (years)	61.2	64.7	65.5	65.5	64.8	63.7	62.4	59.0

Distribution of Charlson indices:								

0	68%	65%	60%	55%	49%	42%	37%	31%
1	20%	17%	16%	15%	13%	10%	6%	4%
2 and 3	10%	17%	22%	27%	35%	45%	54%	60%
4, 5 and 6	2%	2%	2%	3%	3%	3%	4%	5%

Distribution of Admissions per main diagnostic CCS group:								

Neoplasms	13%	20%	25%	31%	40%	52%	65%	72%
Metabolic & shock etc	4%	5%	6%	7%	8%	9%	12%	14%
Heart disease	40%	34%	29%	25%	20%	13%	7%	2%
Respiratory disease	11%	11%	12%	12%	11%	9%	6%	3%
Gastro-intestinal disease	13%	10%	9%	8%	6%	4%	3%	2%
Other 7 main CCS groups	21%	19%	19%	17%	15%	12%	8%	6%

**Admission specific casemix properties**								

Distribution of emergency admissions	43%	37%	39%	37%	34%	28%	18%	7%

Average length of stay (days)	6.8	7.2	7.0	6.5	6.0	5.0	3.4	1.8

## Discussion

(Re)admission frequencies show substantial variations between the hospitals. For example: the overall value of the *re*admission frequency of hospital D equals double the value of hospital B and F. For the main CCS diagnostic group neoplasms, this value for hospital D equals even three times the value of B. We conclude that there are substantial differences in the numbers of readmissions within a given time period between the six hospitals.

Patients with higher admission frequencies bear lower predicted risks per admission, which can be explained by shifts in the casemix. For the patient view however, lowering of risks is only partly predicted by DHM-2008, since the amount of predicted deaths compared to observed deaths turned out to be relatively low for first-and-only admissions and high for readmissions. The corresponding standardised mortality ratio in the patient view is high for class P(m = 1) (127) and low for the other classes (gradually dropping to 35 for P(m > 20)) compared to the overall average of 93. In contrast to this we found that the standardised mortality ratios per n^th ^admission view class are approximately as predicted, fluctuating around 93. DHM-2008 demonstrates quite a fair goodness of fit for the n^th ^admission view classes. This result matches the findings of Jarman [11], where no differences in HSMR were detected by picking n^th ^admissions for any value of n. At the same time however, a comparison of predicted and observed deaths for the patient view classes does not demonstrate a good fit for the model. The question now arises why DHM-2008 should be suited to fit the n^th ^admission view and not be suited to fit the patient view? As we will explain below, different admission frequency classes of the patient view may incur risk differences, not detected by the known adjustment variables. In that case, comparing these classes would commit the constant risk fallacy and establishing a model fit along the lines of the patient view would be preferred to a fit along the lines of the admission view.

We will demonstrate this point by three examples that show mechanisms, lowering risks for higher admission frequencies and having nothing to do with higher quality of care:

*Example 1: *Admission policies may increase the number of readmissions without proportionally increasing real risks. One hospital may systematically combine the diagnosis and treatment into a single admission. Another hospital may have an admission for diagnosis and a second one for treatment, being granted a double predicted risk count, most likely without doubling of real risk, but doubling the expected risk.

*Example 2: *Hospitals may show different treatment practices in the frequency with which chronically ill patients are admitted for the same disease. For example *re*admission frequencies for the treatment of neoplasms in hospitals B and D differ on average by a factor of 3. This difference can be explained by differences in balance between inpatient versus outpatient treatments (late versus early adopters of a trend moving from inpatient toward outpatient treatment, for example [14]). The predicted risk contribution for hospital D may thus be tripled compared to B, but it is not likely that patients of D are being exposed to a tripled real risk. On the contrary, the mortality risk *per patient *stabilizes around 13% if admitted 10 or more times (table 3). Every incremental admission for chemotherapy further increases the denominator of the HSMR, but on average does not increase the observed mortality, the numerator, further.

*Example 3*: Patient referrals to tertiary care, frequently occurring in the Netherlands, may cause differences. The transfer of a patient back and forth most often is an advantage for the referring hospital, as they count a double admission, while the hospital to which the patient is being referred is at a disadvantage because they only count for one admission. On top of this the latter hospital has to deal with the risk of conducting a potentially complicated medical procedure. It is unlikely that a patient under these circumstances will experience a doubling of risk in the referring hospital.

Another plausible mechanism may be hidden in the physical condition of frequently readmitted patients. If these patients are unexpectedly resilient, they will dominate higher admission frequency groups through natural selection and consequently cause lower undetected risks, compared to lower admission frequency groups. If this hypothesis is valid, it might become visible as well in patient specific casemix properties that are known to us such as age and comorbidity (table 6). The average age of frequently admitted patients indeed is decreasing for the highest values of n, indicating a fitter population. The comorbidity is increasing however; a logical consequence of the fact that we applied the notion of readmission for any disease. So patients with more co-morbidity may be admitted more often for the various diseases they suffer from. It also indicates higher vulnerability and in that case would contradict the hypothesis. Although the hypothesis looks appealing, we cannot proof its correctness with the data currently available.

Looking back at the admission history of a frequently admitted patient, the additional risk-lowering factors just described, may have come into play already after the patient's first admission. The n^th ^admission view constitutes a cross-section of various patient view groups, each having their own additional risks. Consequently the n^th ^admission view cannot discriminate additional risk differences and is not suited to be used for readmission adjustment. Instead, we think the variable 'admission frequency' of the patient view should be used for this purpose. Patient views are showing why DHM-2008 predicts too high a risk for a frequently readmitted patient, as illustrated by the following case that we observed:

*Patient × of hospital D contributed 3.1 predicted deaths to the denominator of the HSMR through seven successive admissions within six months. A single patient however can maximally contribute a value of 1 - in case of death - to the numerator of the HSMR*.

Numerous examples alike became available in our study, for example: we found 174 patients in hospital D, each of whom contributed more than 1 predicted death to the denominator of the HSMR due to various readmissions. In total these contributions in hospital D added up to 232 predicted deaths, whereas 'only' 75 deaths were observed in this group. Clearly the number of deaths predicted by DHM-2008 for frequently admitted patients is being overestimated.

**Figure 3:**
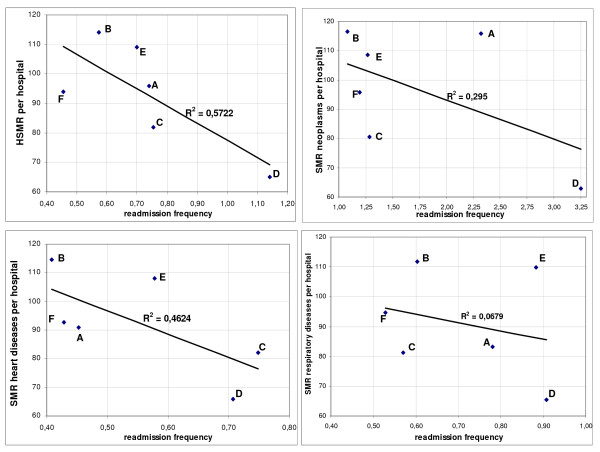
HSMRs and SMRs of neoplasms, heart diseases and respiratory diseases versus readmission frequency *. Linear regression lines are shown.

A final illustration of this phenomenon is shown in figure 3: a scatter diagram where we plotted the HSMRs as well as the SMRs of the three main CCS diagnostic groups that show the largest variations in readmission frequency - neoplasms, heart diseases and respiratory diseases - against the readmission frequency (see also figure 1). In all cases there is a downward trend: higher readmission frequencies corresponding with lower (H)SMRs.

We conclude that there is a significant association between HSMRs and numbers of readmissions per patient.

Since the study involved five consecutive years, we were not able to capture the complete patient view. Patient view sequences that started before 2003 and continued to emerge in the period 2003 - 2007 were truncated. The same happened with sequences that started during the period 2003 - 2007 and continued in the years thereafter. Consequently the picture will never be complete. For each patient being admitted at least twice, we calculated the time elapsed between the date of the first and the date of the last discharge. For 63% of these patients, the time elapsed amounted to less than one year and for 91% less than 3 years, suggesting that the larger part of the effect has been captured.

How can HSMRs be adjusted for the effects of readmissions? For that purpose, an additional adjustment variable 'admission frequency,' as used in this study in the patient view, may be applied. After adjustment, the SMRs of the patient view classes in our study will fluctuate around 93. As a consequence the goodness of fit for the admission view will be lost. However, this does not provide us with a principal problem since the division into admission frequency classes (patient view) is along the lines of identified and distinct risk classes for which adjustment is clearly needed. N^th ^admission classes turn out to be meaningless in terms of risk differentiation.

In particular for the higher admission frequencies, a prolonged measurement period of various years was needed in order to make visible the effects we described. For example the average time which elapsed between the first and last date of discharge for patient view class P(m > 20), amounted to 2.5 years. This does not mean however that having many patients in class P(m > 20), does not have an effect if measured for only one year. Ideally the adjustment for readmission would be based on an 'admission counter' in the file of each patient that is being increased by 1 after every new readmission. Since such counts are not being kept, an approach along the lines of this study, awkward as it might be, will be necessary.

A final remark concerns the following: (frequent) readmissions are sometimes taken to be a proxy indicator for poor quality of care. But instead of working against the hospitals with higher readmission frequencies the current HSMR seems in that case to work in favour of those hospitals, underlining the following statement: If HSMRs in the Netherlands ever will be publically reported and used to compare hospitals, then the issues raised in [4] need to be resolved and, on top of this, adjustment for readmission will be necessary. If HSMRs and particularly SMRs on diagnostic level are used by hospitals as a starting point for quality improvement [2] - a better idea for usage of the HSMR indicator - then adjustment for readmission will be necessary as well, in order to prevent misleading signals to be generated. Hospitals will in that case better be able to avoid falsely acting upon too high SMR values as well as to avoid falsely non-acting upon too low SMR values.

## Conclusions

This study has shown that patients admitted more frequently experience a lower risk of dying per admission. The HSMR model used, detects lower risks for higher admission frequency groups. However the observed mortality of these groups demonstrate the real risks to be even lower, indicating differing risk conditions between the groups. Consequently, comparing admissions of patients from different admission frequency groups, as done by the current HSMR model, commits the constant risk fallacy. The study showed substantial variations in the overall distribution of *re*admission frequencies, up to a factor of 2, between the six hospitals. As a result hospitals with high admission frequencies experience unadjusted decrease of risks and hospitals with low admission frequencies experience unadjusted increase of risks. These misleading outcomes can only be demonstrated by analysis over at least three years, but is in effect every day. As 43% of all admissions in this study concerned readmissions, the impact on the HSMR of some hospitals may be substantial. This finding is relevant for all countries where hospitals use (H)SMRs for monitoring and improving hospital performance. Further research to identify how the effects of readmissions may impact the HSMR in other countries is clearly warranted. The accuracy of the HSMR may improve by taking admission frequency as an additional adjustment parameter.

## Competing interests

The authors declare that they have no competing interests.

## Authors' contributions

All authors have contributed significantly to the conception and design of the study. WB collected and analysed the data and produced the first draft of the manuscript. All authors revised the manuscript critically for important intellectual content and gave final approval of the version to be published.

## Pre-publication history

The pre-publication history for this paper can be accessed here:

http://www.biomedcentral.com/1472-6963/11/57/prepub
